# Crosstalk Between Liver Macrophages and Surrounding Cells in Nonalcoholic Steatohepatitis

**DOI:** 10.3389/fimmu.2020.01169

**Published:** 2020-06-24

**Authors:** Haiou Li, Yunjiao Zhou, Haizhou Wang, Meng Zhang, Peishan Qiu, Mengna Zhang, Ruike Zhang, Qiu Zhao, Jing Liu

**Affiliations:** ^1^Department of Gastroenterology, Zhongnan Hospital of Wuhan University, Wuhan, China; ^2^Hubei Clinical Center, Key Lab of Intestinal and Colorectal Diseases, Wuhan, China

**Keywords:** nonalcoholic steatohepatitis, cellular crosstalk, liver macrophages, liver cells, therapeutic strategies

## Abstract

Nonalcoholic steatohepatitis (NASH), the advanced stage of nonalcoholic fatty liver disease (NAFLD), is emerging as a leading cause of progressive liver fibrosis and end-stage liver disease. Liver macrophages, mainly composed of Kupffer cells (KCs) and monocyte-derived macrophages (MoMFs), play a vital role in NASH progression and regression. Recent advances suggest that cell–cell communication is a fundamental feature of hepatic microenvironment. The reprogramming of cell–cell signaling between macrophages and surrounding cells contributes to the pathogenesis of NASH. In this review, we summarize the current knowledge of NASH regarding the composition of liver macrophages and their communication with surrounding cells, which are composed of hepatocytes, hepatic stellate cells (HSCs), liver sinusoidal endothelial cells (LSECs) and other immune cells. We also discuss the potential therapeutic strategies based on the level of macrophages.

## Introduction

Nonalcoholic fatty liver disease (NAFLD), an increasingly common liver disease worldwide, ranges from relatively benign NAFL to nonalcoholic steatohepatitis (NASH) (1, 2). NASH is strongly associated with progressive liver fibrosis and has further become a major cause of cirrhosis and liver cancer (3). Unlike isolated hepatic steatosis, NASH is characterized as the presence of inflammation, hepatocellular injury, and varying degrees of fibrosis (4). However, the underlying mechanisms involved in pathogenesis of NASH are not fully understood. It was demonstrated that liver macrophages orchestrate both the progression and restoration of NASH (5). Traditionally, liver macrophages mainly comprise liver-resident Kupffer cells (KCs) and circulating monocyte-derived macrophages (MoMFs) (6). The activation of liver macrophages during NASH progression is a dynamic procedure dependent on various stimuli such as cytokines, lipid metabolites, and other signal molecules (7, 8).

Emerging evidence suggests that cellular networks rather than a single cell type modulate NASH progression (9). In conjunction with surrounding cells, liver macrophages can trigger inflammation response, fibrogenesis, vascular remodeling, and so forth. In the development of NASH, hepatocytes contribute to KC activation and MoMF recruitment *via* multiple signal molecules such as damage-associated molecular patterns (DAMPs), extracellular vesicles (EVs), and harmful lipids (5). In response to those signals, activated macrophages also signal back to modulate hepatocyte fate. Besides, those activated macrophages further mediate the activation of hepatic stellate cells (HSCs) *via* producing cytokines and chemokines, including transforming growth factor-β (TGFβ), interleukin-1β (IL-1β), platelet—derived growth factor (PDGF) receptor, and CC-chemokine ligand 2 (CCL2) (10). Moreover, liver macrophages influence the biological functions of liver sinusoidal endothelial cells (LSECs) and other immune cells (11, 12). In turn, those surrounding cells can stimulate liver macrophages during NASH progression (13, 14). Understanding the intercellular crosstalk between liver macrophages and their surrounding cells is critical for developing novel therapeutic interventions based on the level of macrophages.

In this review, we summarize the intercellular signaling between liver macrophages and surrounding cells involved in NASH development. The potential macrophage-targeted therapeutic strategies for NASH are also discussed.

## The Composition of Liver Macrophages in Nonalcoholic Steatohepatitis

Liver macrophage populations comprise different subsets of cells. In particular, KCs and freshly recruited MoMFs are important mediators of liver inflammation, fibrogenesis, and fibrinolysis in the development of NASH (15, 16). In mice, circulating monocytes were divided into two main subsets: lymphocyte antigen 6C high (Ly-6C^hi^) and Ly-6C low (Ly-6C^lo^) expressing monocytes. It was demonstrated that the hepatic infiltration of Ly-6C^hi^ monocytes occurred early in murine NASH models and patients with NASH (16, 17). Those monocytes gave rise to phenotypically distinct populations of MoMFs upon external stimulus. Briefly, KCs and MoMFs could be differentiated toward either a classic proinflammatory phenotype (M1 macrophages) or an alternative anti-inflammatory phenotype (M2 macrophages) *in vitro* (18). The M1 macrophages produced proinflammatory cytokines such as tumor necrosis factor α (TNFα), IL-1β, CCL2, and CCL5. In contrast, M2 macrophages secreted a distinct set of mediators including IL-13, IL-10, IL-4, and TGFβ (19). It was noted that KCs and MoMFs in NASH liver exhibited a notable shift toward a proinflammatory phenotype on the basis of their gene expression signatures at the single-cell level (20).

In a recent single-cell RNA sequencing (scRNA-seq) study, two distinct subpopulations of liver macrophages are exhibited in western diet (WD)-induced NASH models in mice, including MoMFs with high lysozyme 2 (Lyz2) expression and KCs with high C-type lectin domain family 4 member F (Clec4f) expression (21). Besides, those MoMFs segregated into three subtypes owing to their striking heterogeneity (21). Furthermore, a NASH-specific macrophage population, marked by high expression of triggering receptors expressed on myeloid cells 2 (Trem2), was observed in NASH livers of both mice and humans, termed NASH-associated macrophages (NAMs) (20). Consistently, another scRNA-seq study identified a pathogenic subpopulation of TREM2^+^CD9^+^ macrophages in the fibrotic niche of human liver with NASH, named scar-associated macrophages (SAMacs). The expansion of SAMacs was positively correlated with the degree of NASH-induced liver fibrosis (22). More studies are needed to understand the ontology of hepatic macrophage subpopulations in NASH.

## Intercellular Crosstalk of Liver Macrophages in Nonalcoholic Steatohepatitis

**Figure 1:**
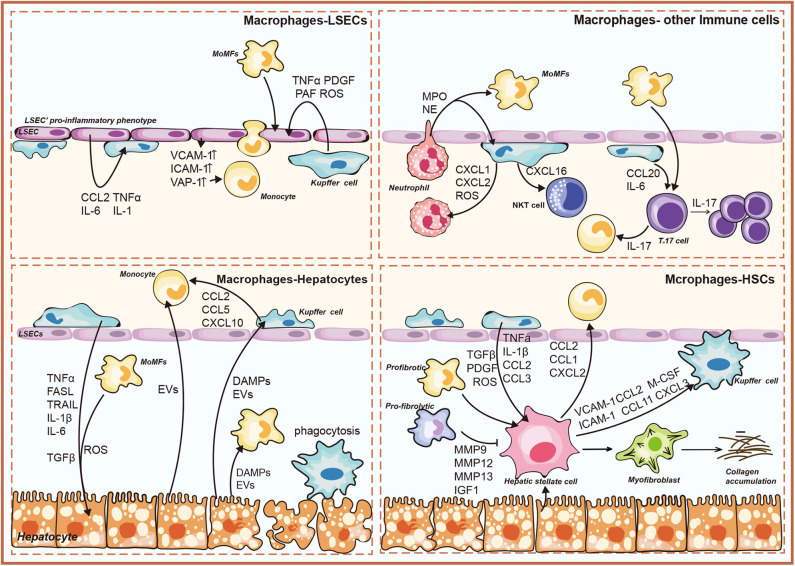
Overview of liver macrophage-related intercellular signaling in nonalcoholic steatohepatitis (NASH). The illustration consists of four groups, as follows: liver macrophages–hepatocytes; liver macrophages–hepatic stellate cells (HSCs); liver macrophages–liver sinusoidal endothelial cells (LSECs); liver macrophages–immune cells. DAMPs, damage-associated molecular patterns; EVs, extracellular vesicles; TNFα, tumor necrosis factor α; TRAIL, TNF-related apoptosis-inducing ligand; FasL, Fas ligand; ROS, reactive oxygen species; CCL, chemokine (C-C) motif ligand; CXCL, chemokine (C-X-C motif) ligand; IL, interleukin; MMP, matrix metalloproteinase; IGF1, insulin-like growth factor 1; TGFβ, transforming growth factor-β; M-CSF, macrophage colony-stimulating factor; PDGF, platelet-derived growth factor; PAF, platelet-activating factor; ICAM-1, intercellular adhesion molecule-1; VCAM-1, vascular cell adhesion molecule-1; VAP-1, vascular adhesion protein-1; MPO, myeloperoxidase; NO, nitric oxide; IFNγ, interferon γ.

The growing consensus is that cell–cell communication within liver represents a key aspect that leads to the progression toward NASH (9). The anatomical location of liver macrophages allows them to interact with several liver resident cells and circulating immune cells (23). Histologically, the clusters of KCs were characterized as microgranulomas, and those with lipid droplets were characterized as lipogranulomas in human NAFLD/NASH (24–26). A unique histological structure, where activated macrophages aggregated around hepatocytes with large lipid droplets, was detected in the murine NASH models and patients with NASH, termed hepatic crown-like structures (hCLS) (27). Conversely, activated KCs were not shown to form hCLS in patients and mice with simple steatosis (28). This section focuses on liver macrophage-related crosstalk in NASH (Figure 1).

## Interaction Between Liver Macrophages and Hepatocytes

Lipotoxicity is characterized as a key feature that differentiated NASH from isolated steatosis (29, 30). Various lipotoxic compounds (e.g., free cholesterol, ceramides, and saturated fatty acids) induce metabolic stress, oxidative stress, and endoplasmic reticulum-related stress in hepatocytes, resulting in hepatocyte injury and death (31). Hepatocyte stress and death cause the release of their cellular contents into extracellular space, which contributes to macrophage activation (29).

### Kupffer Cells-Hepatocytes

The DAMPs, such as cytosolic proteins, purine nucleotides, and mitochondrial compounds, primarily acted on pattern recognition receptors (PRRs) to promote inflammatory responses of KCs (32). High mobility group box-1 (HMGB1) was a widely studied DAMP that induced cytokine release of macrophages (33). It bound toll-like receptor 4 (TLR4) to induce nuclear factor (NF)-κB translocation and TNFα release in KCs (34). Besides, the mitochondrial DNA (mtDNA) released from damaged hepatocytes activated TLR9 on KCs to promote inflammatory response (35). Recently, it was reported that the mtDNA was recognized by the stimulator of IFN genes (STING) in KCs to induce TNFα and IL-6 production under lipid overload (36). Adenosine triphosphate (ATP) was also released into extracellular space from injured hepatocytes. Being sensed by P2X purinoceptor 7 (P2X7) receptor on KCs, ATP could medicate the induction of NLR family pyrin domain-containing 3 (NLRP3) inflammasome and the consequent production of proinflammatory cytokines (37, 38).

**Figure 2:**
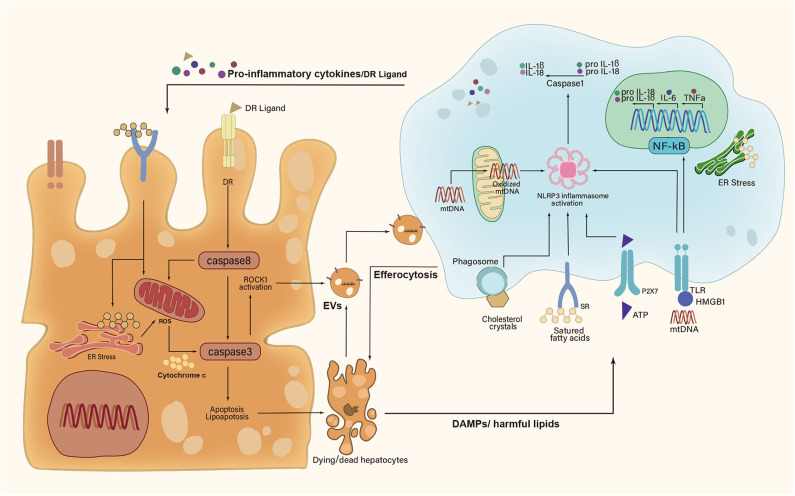
A feed-forward regulatory loop between lipotoxic hepatocytes and Kupffer cells. Upon metabolic stress, dying and dead hepatocytes release damage-associated molecular patterns (DAMPs), extracellular vesicles (EVs), and harmful lipids to activate Kupffer cells (KCs). In turn, activated KCs secrete proinflammatory cytokines and death receptor (DR) ligands to aggravate hepatocyte damage. However, KCs can remove apoptotic hepatocytes *via* efferocytosis. IL-1β, interleukin-1β; IL-6, interleukin-6; IL-18, interleukin-18; TNFα, tumor necrosis factor α; HMGB1, high mobility group box-1; ATP, adenosine triphosphate; mtDNA, mitochondrial DNA; ROCK1, rho-associated, coiled-coil containing protein kinase 1; NF-κB, nuclear factor-κB; NLRP3, NLR family pyrin domain-containing 3; ER, endoplasmic reticulum; ROS, reactive oxygen species; P2X7, P2X purinoceptor 7; TLR, toll-like receptor; SR, scavenger receptor.

Recent studies implicated lipotoxic hepatocyte-derived EVs (LPC-EVs) in mediating cell–cell communication by transferring various cargos (39). Apoptotic bodies formed by apoptotic hepatocytes fall in the category of EVs. Engulfment of apoptotic bodies by KCs promoted the production of TNFα, TNF-related apoptosis-inducing ligand (TRAIL), and Fas ligand (FasL) (40). These death receptor (DR) ligands further induced hepatocyte apoptosis in a feed-forward loop (Figure 2). Moreover, KCs were shown to aggregate around dead hepatocytes to form hCLSs. Specifically, the cholesterol crystals within remnant lipid droplets of dead hepatocytes were processed by KCs, which then activated the NLRP3 inflammasome in KCs, causing proinflammatory cytokines production (41). In this line, NLRP3 inflammasome blockade improved cholesterol crystal-derived inflammation and fibrosis in experimental NASH (42).

In response to those signals sent by hepatocytes, KCs also signaled back to the hepatocytes and regulated their fate (43–46) (Figure 2). Firstly, activated KCs exerted actions *via* producing cytokines (e.g., IL-6, TNFα, and IL-1β) (47). TNFα allowed for the activation of caspase-8 in hepatocytes by binding to TNF receptor 1 (TNFR1), which not only triggered apoptotic caspase cascade directly but also induced mitochondrial dysfunction to amplify the signals indirectly (8, 48). In addition, KC-derived IL-1β signaling was associated with *de novo* lipogenesis in hepatocytes and promoted hepatic lipid deposition (49–51). IL-6 contributed to insulin resistance in hepatocytes by disrupting key steps in the insulin signal transduction (52). Additionally, KCs were shown to remove apoptotic hepatocytes *via* efferocytosis (9). The efferocytic clearance of dead hepatocytes prevents the release of DAMPs and subsequent DAMP-mediated inflammation. Efferocytosis could be triggered by a series of “eat-me” signals from apoptotic hepatocytes (53). The well-studied “eat-me” signal was the presence of phosphatidylserine (PtdSer) on the outer leaflet of the cell membrane during apoptosis (54). KCs were thought to be the most important hepatic efferocytes with the expression of several different PtdSer receptors, such as T cell immunoglobulin, mucin domain-containing molecule 3 (Tim3), Tim4, macrophage c-mer tyrosine kinase (MerTK), stabilin-1, and stabilin-2 (53). Strikingly, both Tim3 and Tim4 were overexpressed in all detected liver macrophage subsets in methionine- and choline-deficient diet (MCD)-induced NASH mice (55, 56). Their absence led to increased production of reactive oxygen species (ROS), IL-1β, and IL-18 in macrophages, concomitant with the aggravation of steatosis and liver fibrosis (55, 56). Further studies are urgently needed to explore that how those PtdSer receptors participate in efferocytosis mechanisms of macrophages during NASH development.

### Monocyte-Derived Macrophages-Hepatocytes

NASH-induced hepatocyte damage recruited MoMFs indirectly by stimulating KCs to release proinflammatory chemokines including CCL2, CCL5, and CXCL10 (57). Lipotoxic hepatocytes also release EVs to induce the hepatic recruitment of MoMFs. TRAIL-enriched LPC-EVs induced the expression of IL-1β and IL-6 *via* NF-κB activation in mouse bone marrow-derived macrophages (58). Ceramide and chemokine (C-X-C motif) ligand 10 (CXCL10) within EVs contributed to MoMF recruitment to the liver *via* activating macrophage chemotaxis (59, 60). Besides, integrin β (ITGβ) enriched LPC-EVs mediated monocyte adhesion to LSECs, an essential step for hepatic recruitment of MoMFs in murine NASH (61).

## Interaction Between Liver Macrophages and Hepatic Stellate Cells

Studies revealed that macrophages were key regulators in the pathogenesis of NASH-driven fibrosis (9, 62). Similarly, therapeutic inhibition of macrophage infiltration accelerated liver fibrosis regression in murine NASH (16, 63, 64). Besides, macrophages aggregated to form hCLS where they could interact with HSCs (27). The hCLS was located close to fibrogenic lesions and the number of hCLS significantly linked to the extent of liver fibrosis (27, 41). In turn, activated HSCs were shown to regulate macrophage accumulation and proliferation through paracrine effects (65). Moreover, a scRNA-seq analysis showed that activated HSCs were implicated in modulating the functions of macrophages *via* a series of stellakines (e.g., CCL2, CCL11, and CXCL2) in murine NASH models (20).

### Kupffer Cells–Hepatic Stellate Cells

On the molecular level, KCs regulated HSC activation by producing cytokines and chemokines such as TGFβ, PDGF, TNFα, and IL-1β (10). KC-derived TGFβ promoted HSC differentiation into a profibrogenic phenotype, concomitant with increased collagen and α-smooth muscle actin expression (66). Recently, Cai et al. proved that the MerTK signaling in KCs promoted HSC activation and liver fibrosis in NASH mice *via* TGFβ1 production (67). Moreover, TGFβ induced oxidative DNA damage in HSCs through downregulation of cytoglobin (68). Besides, in murine NASH models, the enhancement of TNFα signaling following KC activation facilitated HSC survival *via* activating the NF-κB pathway in HSCs (69, 70). Activated KCs caused the HSC migration and recruitment through the secretion of CCL2 and CCL5 (71, 72). On the other hand, the HSC-derived chemokines that included CCL2 and macrophage colony-stimulating factor (M-CSF) further activated KCs, amplifying the inflammatory response (73). In response to lipopolysaccharide (LPS), HSCs secreted intercellular adhesion molecule-1 (ICAM-1), vascular cell adhesion molecule-1 (VCAM-1), and E-selectin to induce KC migration (74). The underlying mechanisms governing this process have not been fully elucidated.

### Monocyte-Derived Macrophages–Hepatic Stellate Cells

Infiltrating MoMFs are divided into two major subsets: Ly-6C^hi^ macrophages and Ly-6C^lo^ macrophages. Similar to KCs, proinflammatory Ly-6C^hi^ macrophages activated HSCs by secreting TGFβ, IL-1β, PDGF, and CCL2, enhancing the fibrotic response. Recently, Ramachandran P et al. demonstrated that the TREM2^+^CD9^+^ SAMacs, differentiating from circulating monocytes, performed a profibrogenic characteristic with multiple profibrogenic genes expression (22). Of note, during the regression stage, the pro-restorative Ly-6C^lo^ macrophages promoted HSC apoptosis and accelerated extracellular matrix degradation by increasing the expression of matrix metalloproteinase 9 (MMP9), MMP12, MMP13, and insulin-like growth factor 1 (IGF1) (75, 76). This pro-restorative subpopulation also expressed chemokine (C-X3-C motif) receptor 1 (CX3CR1), and its ligand CX3C ligand 1 (CX3CL1) was mainly expressed by HSCs (77). The CX3CL1–CX3CR1 interaction negatively regulated the inflammatory properties in macrophages (78).

## Interaction Between Liver Macrophages and Liver Sinusoidal Endothelial Cells

LSECs constituted a unique vascular bed with fenestrae in liver and interacted directly with the immune cells and antigens in the blood flow (79). Monocyte's adhesion to LSECs is a crucial step for inflammation response in NASH, which verified the “gatekeeper” role of LSECs in the progression from simple steatosis to NASH (80).

### Kupffer Cells–Liver Sinusoidal Endothelial Cells

At the early stage of NAFLD, LSECs exhibited an anti-inflammatory property by inhibiting KC activation and monocyte migration (81, 82). At the stage of NASH, LSEC capillarization happened, and capillarized LSECs were necessary for activation of KCs (83). LSECs acquired a proinflammatory phenotype to produce proinflammatory mediators, leading to KC activation (14). Activated KCs were shown to be involved in angiogenesis through the secretion of ROS and cytokines including TNFα, PDGF, and platelet-activating factor (PAF) (84).

### Monocyte-Derived Macrophages–Liver Sinusoidal Endothelial Cells

In NASH, the proinflammatory phenotype of LSECs increased proinflammatory chemokine CCL2 to facilitate hepatic recruitment of monocytes (14). Moreover, in mice models of NASH, the overexpression of adhesion molecules ICAM-1, VCAM-1, and vascular adhesion protein-1 (VAP-1) in LSECs were critical for the adhesion and transmigration of monocytes to amplify local inflammatory response (14, 85, 86). Little is known about the pathophysiological roles of MoMFs toward LSECs in NASH.

## Interaction Between Liver Macrophages and Other Immune Cells

### Kupffer Cells–Other Immune Cells

The interactions of immune cells in homeostasis and disease have been reviewed in detail elsewhere (87). Firstly, KCs contribute to the hepatic infiltration of neutrophils in NASH. The inflammatory activation of KCs resulted in the production of chemokines (e.g., CXCL1, CXCL2, and CXCL8) and ROS, which stimulated neutrophil recruitment to expanded inflammation (44, 72). Hepatic neutrophil content and neutrophil elastase (NE) activity were significantly increased in high-fat diet (HFD)-fed mice. NE treatment caused the proinflammatory markers of macrophages to largely increase (88). Neutrophil-derived myeloperoxidase (MPO) was also associated with the formation of hCLS in NASH (89). Besides, activated KCs promote natural killer T (NKT) cell over-activation and subsequent deficiency in the pathogenesis of NAFLD (90). KC-derived IL-12 was associated with the reduced numbers of hepatic NKT cells in hepatosteatosis (91). Conversely, Syn et al. described that NKT cells were associated with NASH-related fibrosis (92). CXCL16 secreted by KCs triggered the hepatic accumulation of CXCR6^+^ NKT cells, thereby accentuating liver inflammation and fibrosis in murine liver (93).

### Monocyte-Derived Macrophages–Other Immune Cells

A proinflammatory phenotype of macrophages showed a close relationship with diverse T-cell subsets by secreting IL-6, TNFα, IL-1β, IL-12, and IL-23 in the pathogenesis of NAFLD (12). Although these cytokines are well-established drivers of T-cell differentiation, their roles in controlling T-cell differentiation in NASH are not fully understood (87). T helper type 17 (Th17) cells and their production of IL-17 facilitated the transition from simple steatosis to steatohepatitis in NAFLD (94). They favored the further activation of monocytes, leading to the release of proinflammatory cytokines that, in turn, amplified liver inflammation (95).

## Macrophage-Targeted Therapeutic Interventions in Nonalcoholic Steatohepatitis

Currently, there are still no Food and Drug Administration (FDA)-approved effective drugs for NASH despite its high prevalence. Owing to their critical roles in NASH, liver macrophages are emphasized as attractive targets for NASH treatment. Specifically, there are some options that exert potential therapeutic effects by regulating cell–cell communication in NASH.

Because the recruited MoMFs widely interact with resident cells, interfering with recruiting signals would disrupt intercellular communication at the level of macrophages. Cenicriviroc (CVC), a dual CCR2/5 antagonist, efficiently reduced the hepatic recruitment of MoMFs that ameliorated hepatic inflammation and fibrosis in NASH mice models (64). This drug was evaluated in a phase II clinical trial in NASH patients and was found to be effective in reducing fibrosis after CVC administration (96). The RNA-aptamer molecule mNOX-E36 also relieved steatohepatitis and accelerated regression of liver fibrosis in experimental mouse models *via* antagonizing CCL2 (63). Maraviroc, a CCL5 inhibitor, ameliorated hepatic steatosis in HFD-induced NAFLD in mice (97). Moreover, monocyte's adhesion to LSECs is an essential step for hepatic recruitment of MoMFs. The VAP-1 inhibitor, also called amine oxidase copper containing three (AOC3) inhibitor, decreased inflammatory cell recruitment and reduced fibrosis (85). This drug was tested in a phase II trial in patients with NASH, but it was discontinued owing to the risk of drug interactions in NASH patients (98).

Another potential NASH treatment is to regulate KC activation. Because hepatocyte-derived DAMPs trigger the sterile inflammatory response of KCs by acting on PRRs, targeting released DAMPs or PRRs can inhibit KC activation, thus ameliorating liver inflammation (99). HMGB1 neutralizing antibodies and PRR antagonists (e.g., TLR2, TLR3, and TLR4 antagonists) were shown to attenuate liver inflammation in murine models (100, 101). Targeting macrophage-derived profibrogenic molecules may be promising to improve NASH fibrosis. Galectin-3 is a profibrogenic protein that is highly expressed in macrophages surrounding lipotoxic hepatocytes. Treatment with galectin-3 inhibitor (GR-MD-02) markedly improved fibrosis in a murine model of NASH (102). A phase IIb trial showed that GR-MD-02 reduced the hepatic-portal vein pressure gradient in patients with NASH cirrhosis (103).

Another potential option is regulating intracellular pathways in macrophages, which has been reviewed elsewhere (5). Notably, non-coding RNAs (ncRNAs) offered new possibilities in developing therapeutic strategies for NASH on the basis of the level of macrophages (104, 105). For instance, in murine fibrotic NASH models, treatment with miR-223-3p mimic ameliorated activation of HSCs and fibrosis development through its NLRP3-targeted effect in KCs (106). In addition, miR-146b acted as a promising approach to attenuate HFD-induced NASH in mice by directly targeting the IL-1 receptor-associated kinase 1 and TNFR-associated factor 6 in macrophages, resulting in suppression of TNFα and IL-6 (107). A cell-specific delivery system with efficiency and safety is essential for the clinical application of those miRNAs.

## Conclusion and Future Perspectives

Multiple studies have shown that liver macrophages play a central role in the progression and regression of NASH. They sense various external signals and act as key mediators of hepatic inflammation. Importantly, owing to their strategic location, liver macrophages can interact with different cells such as hepatocytes, HSCs, and LSECs. However, there are several issues that need to be addressed. Firstly, most of the observed interactive effects are in specific cytokine-dependent manner. The core intracellular pathways of macrophages in mediating intercellular signaling in NASH are still unclear, which points out a future research goal. Secondly, owing to their striking heterogeneity, more studies are needed to reveal the complex cell–cell communication network based on the large spectrum of macrophage phenotypes. In addition, most findings from murine models are insufficient to reflect the complex cellular networks during NASH progression in humans. Further exploration of the macrophage function in human NASH liver is warranted.

Moreover, liver macrophages are identified as attractive targets for NASH treatment. As described in this review, some signaling pathways that mediated cellular crosstalk are potentially druggable. Besides, the rapid advancement in nanomedicine allows for targeted delivery of drugs to macrophages, such as miRNA mimic. Taken together, deciphering macrophage function and their role in intercellular signaling network will facilitate the design of novel targeted therapies to treat NASH.

## Author Contributions

HL and YZ searched the literature and wrote the manuscript. HW prepared the figures. MengZ, PQ, MengnaZ, and RZ carefully checked the manuscript and helped to improve paragraphs. QZ and JL designed and revised the manuscript. All authors contributed to the article and approved the submitted version.

## Conflict of Interest

The authors declare that the research was conducted in the absence of any commercial or financial relationships that could be construed as a potential conflict of interest.
